# Implementing Statewide Newborn Screening for New Disorders: U.S. Program Experiences

**DOI:** 10.3390/ijns6020035

**Published:** 2020-04-30

**Authors:** Yvonne Kellar-Guenther, Sarah McKasson, Kshea Hale, Sikha Singh, Marci K. Sontag, Jelili Ojodu

**Affiliations:** 1 Center for Public Health Innovation, CI International, Littleton, CO 80120, USA; msontag@ciinternational.com; 2 Department of Community and Behavioral Health, Colorado School of Public Health, University of Colorado, Anschutz Medical Campus, Aurora, CO 80045, USA; 3 Department of Epidemiology, Colorado School of Public Health, University of Colorado, Anschutz Medical Campus, Aurora, CO 80045, USA; sarah.mckasson@cuanschutz.edu; 4 Association of Public Health Laboratories, Silver Spring, MD 20910, USA; kshea.hale@aphl.org (K.H.); sikha.singh@aphl.org (S.S.); jelili.ojodu@aphl.org (J.O.)

**Keywords:** newborn screening, new conditions, Pompe, Mucopolysaccharidosis type I (MPS I), X-linked adrenoleukodystrophy (ALD), Spinal Muscular Atrophy, newborn screening readiness

## Abstract

Data were collected from 39 newborn screening (NBS) programs to provide insight into the time and factors required for implementing statewide screening for Pompe, Mucopolysaccharidosis type I (MPS I), adrenoleukodystrophy (ALD), and Spinal Muscular Atrophy (SMA). Newborn screening program readiness to screen statewide for a condition was assessed using four phases: (1) approval to screen; (2) laboratory, follow-up, and information technology capabilities; (3) education; and (4) implementation of statewide newborn screening. Seventeen states (43.6%) reached statewide implementation for at least one new disorder. Those states reported that it took 28 months to implement statewide screening for Pompe and MPS I, 30.5 months for ALD, and 20 months for SMA. Using survival curve analysis to account for states still in progress, the estimated median time to statewide screening increased to 75 months for Pompe and 66 months for MPS I. When looking at how long each readiness component took to complete, laboratory readiness was one of the lengthier processes, taking about 39 months. Collaboration with other NBS programs and hiring were the most frequently mentioned facilitators to implementing newborn screening. Staffing or inability to hire both laboratory and follow-up staff was the most frequently mentioned barrier.

## 1. Introduction

Newborn screening (NBS) has played an important role in identifying infants with specific genetic conditions that are asymptomatic at birth so they can receive faster medical intervention [1,2]. The Recommended Uniform Screening Panel (RUSP) lists disorders that have passed scientific review and are recommended for universal screening in the U.S. The RUSP was based on a report authored by the American College of Medical Genetics and Genomics (ACMG) and endorsed by the U.S. Secretary of Health in 2010 [3,4]. The RUSP was created in response to a recommendation from the American Academy of Pediatricians Newborn Screening Task Force to create uniformity in NBS throughout the U.S. as well as a process for government, professionals, and consumers to nominate a disorder to be considered by all state NBS programs [3,5]. Although the RUSP provides recommendations and not requirements, most states look to the RUSP when determining whether to screen for a disorder. California and Pennsylvania, however, are required to screen for a disorder within two years once it has been added to the RUSP [6,7]. 

The RUSP initially included 29 core conditions and has since expanded to include 35 core conditions as of 2020 (https://www.hrsa.gov/advisory-committees/heritable-disorders/rusp/index.html, accessed 16 September 2019). A core condition is a disorder the newborn screen is designed to identify. The process of adding conditions to the RUSP has evolved (https://www.hrsa.gov/advisory-committees/heritable-disorders/rusp/nominate.html) but has always required an evidence review process followed by a vote by the Advisory Committee on Heritable Disorders in Newborn and Children (ACHDNC) [4,8]. The consideration of new disorders for the RUSP stems from advances in treatment options, changes in testing technology, and public comments [9]. The ACHDNC determines if a condition should be added to the RUSP by assessing if (1) detection through the newborn screen is beneficial to the health outcome of the child, (2) there are low harms associated with testing for the condition, (3) there is an effective and efficacious screening test, (4) treatment is available for newborns or infants diagnosed with the disorder, and (5) it is determined that NBS programs have the capability and it is feasible for them to provide comprehensive screening based on feedback from newborn screening state programs [4].

The last criterion, NBS program capability and feasibility, is captured through a Public Health Impact Assessments (PHIA), conducted by Duke University in coordination with the Association of Public Health Laboratories (APHL) every time a new condition is reviewed for potential addition to the RUSP. The PHIA includes a self-reported determination of readiness by the NBS program to obtain authority to screen, to conduct laboratory testing, to interpret and report results, to track blood spot specimens, and to have coordinated systems for diagnostic evaluation [4]. Since the addition of revised recommendation criteria to be used by ACHDNC in 2013 to include the PHIA [10], four new disorders have been adopted by ACHDNC for addition to the RUSP—Pompe disease, added 2 March 2015; Mucopolysaccharidosis type I (MPS I), added 16 February 2016; X-linked adrenoleukodystrophy (ALD), added 16 February 2016; and Spinal Muscular Atrophy (SMA), added 2 July 2018 [6,11,12,13]. The majority of states that responded to the PHIAs for all four conditions indicated they were in a state of “developmental readiness,” meaning they believed they could begin screening in one to three years after approval from their state leadership to screen and obtain approval for the funding [12,13]. While these data may offer some insight during the consideration process for the proposed disorder, they are estimates of the time needed. There are no systematically collected national data that provide information on the time elapsed when implementing a new disorder.

Understanding the time taken to begin screening for a new disorder can provide awareness about which parts of the implementation process are the most time-consuming, allowing for a broader discussion of what support could be most beneficial to NBS programs as they work to expand the number of disorders screened for their communities. The goal of this research is to examine how long it takes NBS programs to begin implementation of statewide screening for Pompe, MPS I, ALD, and/or SMA. Additionally, we examine how long different readiness phases take to complete using prospective data to gain insight into the needs of NBS programs.

## 2. Material and Methods

### 2.1. NewSTEPs and State Implementation of New Disorders

The Association of Public Health Laboratories (APHL) [14] received funding from the Health Resources and Services Adminstration (HRSA # UG9MC30369) to support state newborn screening programs to implement three disorders recently added to the RUSP: Pompe, MPS I, and ALD. The funding was also used to build upon the technical assistance provided through its Newborn Screening Technical assistance and Evaluation Program (NewSTEPs). Through a competitive application process, 16 state NBS programs were awarded funding to implement at least one of the new disorders. All U.S. state and territory NBS programs were invited to an in-person New Disorder Annual Conferences to discuss the implementation of the new disorders.

### 2.2. Data Collection

This project is program improvement and was not deemed to be human subjects research.

#### 2.2.1. New Disorder Readiness Tool

The Readiness Tool was created in October 2016 to systematically capture data on the length of time to complete implementation activities leading to statewide screening for a new disorder, using the PHIA readiness components [4] as a guide. The Readiness Tool is broken into four stages: (1) authority to screen and authority for funding to screen, (2) program readiness (laboratory, follow-up, and information technology (IT)), (3) education, and (4) full implementation; each phase entails between 3 and 33 activities. The tool was refined following discussion by the NewSTEPs Steering Committee and disseminated in March 2017. 

The Readiness Tool was implemented using REDCap electronic data capture tools [15,16], hosted at the University of Colorado, Colorado School of Public Health, with a separate module for each disorder and secure logins for NBS program personnel. For each implementation activity, programs were asked to select their status (“Not Started”, “Started”, “Completed/Implemented” and “Not Applicable”). For any activity in the started or completed phase, NBS programs were asked to provide corresponding dates. If the exact date was unknown, respondents were asked to use the first of the month. Laboratory and follow-up staff were encouraged to complete the tool together to ensure the accuracy of the timeline. 

NBS programs (awardees) that were funded by the NewSTEPs New Disorders Project (HRSA # UG9MC30369) were asked to update The Readiness Tool at least five times over three years. These dates were April 2017, April 2018, August 2018, February 2019, and August 2019. Additionally, NBS staff who attended the New Disorders Annual Conference were asked to provide data prior to the meeting even if their states were not funded by the project. Staff reached out to programs to discuss inconsistencies or questions found in the data and updates were made in REDCap. All states who provided data were asked to update and verify their data for this publication by 31 August 2019.

#### 2.2.2. New Disorder Awardee Reporting and Interviews

The 16 NBS programs that were awarded funding through the NewSTEPs New Disorders project were asked to identify their successes, challenges, and needs as part of their annual reporting requirements. Twelve states provided written reports for the first year of the program, and 14 provided reports in the second year. Two NBS programs provided verbal reports via telephone. 

Additionally, seven programs were also asked to participate in key informant interviews. The interviewees were chosen because their readiness tool data submissions revealed that their programs’ time to completion of the various stages of implementation fell either among the two most time-efficient or the two most time-intensive. The goal of these interviews was to provide a deeper understanding of the variation in time during the readiness process.

### 2.3. Data Analysis

#### 2.3.1. Descriptive Statistics

The time elapsed between the start date and end date was calculated for both phase and activity completion; programs that did not provide both dates were excluded from the corresponding analysis. Authorization to screen was considered complete when an NBS program provided a date for “mandate or approval to screen”, and approval for funding was based on the date when the fee increase was implemented. The time elapsed between these completion dates and the date of the first activity within these approval steps was calculated. NBS programs were asked to check a box when laboratory, follow-up, and IT readiness was complete. In these cases, the earliest start date and latest completion dates for lab, follow-up, and IT were used to compute the date difference. Education readiness was considered complete when states provided a date for the distribution of the materials. Finally, for time elapsed to implement statewide screening, differences were calculated by time elapsed between the earliest start date entered across all activities and the implementation date. Median time differences are reported as observed median unless otherwise indicated.

#### 2.3.2. Survival Analysis

An estimation of implementation time of statewide screening and phase completion was assessed with a Kaplan–Meier time-to-event analysis. This analysis included data from all NBS programs that provided a start date for a phase. To predict the median time for implementation of statewide screening and phase completion, a Kaplan–Meier survival analysis using SAS™ PROC LIFETEST was conducted. The time of entry was defined as the start date of the first activity. NBS programs that had not achieved full implementation were censored at the end of data collection (31 August 2019); programs that had not started an activity or selected “not applicable” were excluded from the analysis. The findings for the survival analysis are reported as predicted median time. SMA was not included in the survival analysis as not enough time had elapsed from its inclusion on the RUSP to the end of data collection to allow for accurate estimates.

#### 2.3.3. Qualitative Analysis

The awardee report narratives from all 16 programs were reviewed and coded to identify barriers and facilitators to readiness by two trained staff. One staff independently coded the narratives and identified barriers and facilitators. The second coder added codes that were missing and highlighted any disagreements. The final coding was agreed upon by discussion with the two coders. The interviews were coded for themes using a coding scheme developed through a review of the report narratives. Themes that were mentioned by at least three states are reported.

## 3. Findings

Thirty-nine U.S. NBS programs provided data on the New Disorder Readiness Tool. Two (5.1%) of these programs served as Peer Resource Network Centers (PRNCs), providing technical assistance to awardees; 16 (41.1%) received financial support from the NewSTEPs New Disorders project to work towards statewide implementation for Pompe, MPS I, and ALD; and 21 (53.8%) attended the New Disorders Annual Conference but did not receive funding from NewSTEPs. As of 31 August 2019, 17 of the 39 (43.6%) NBS programs who provided data reached statewide implementation for at least one new disorder. Table 1 shows how many states had implemented statewide screening, were in progress toward implementation, or had not yet started implementation activities for each new disorder.

### 3.1. Time to Implementation of Statewide Screening

Among the 17 participating programs that fully implemented a disorder prior to 31 August 2019, the median observed time from the earliest start date to the implementation date for statewide screening differed by 10.5 months amongst the new disorders. It took a median of 28 months to implement statewide screening for Pompe and MPS I (*n* = 11 and 13 respectively), 30.5 months for ALD (*n* = 8), and 20 months for SMA (*n* = 5) (Figure S1).

**Figure 1:**
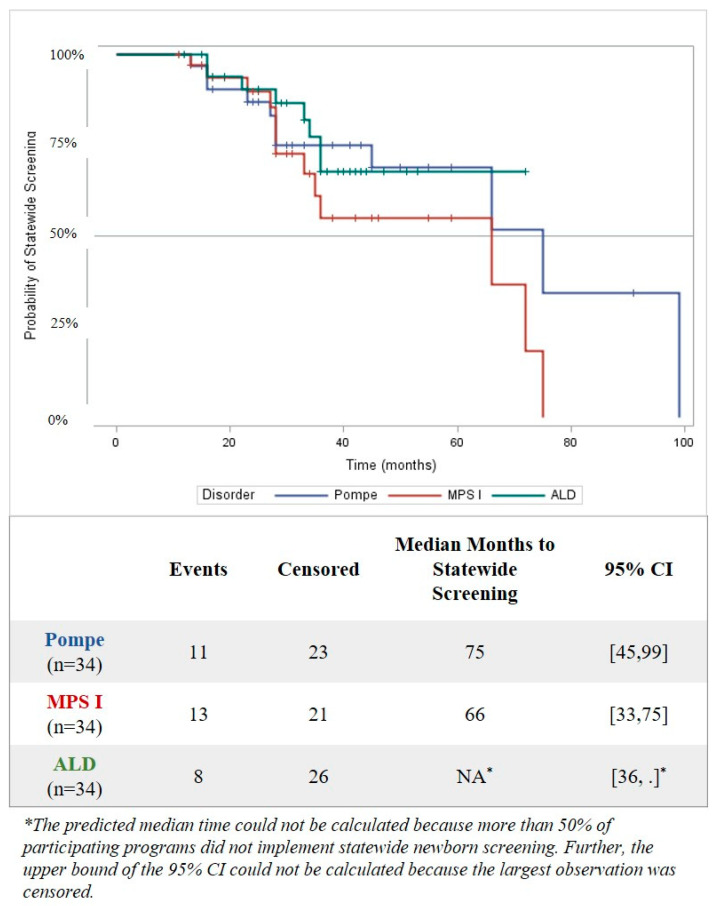
Elapsed Time from First Programmatic Activity to Implementation of Statewide Newborn Screening for Pompe, Mucopolysaccharidosis type I (MPS I), and adrenoleukodystrophy (ALD) using Kaplan–Meier Survival Analysis, as of August 2019. Programs without a recorded date of implementation by 1 August 2019 were considered censored.

Due to more than half of the states in this study having not yet started screening for Pompe, MPS I, ALD, and SMA (Table 1), a survival analysis was used to predict how much time would be needed to reach statewide implementation for states still “in progress.” The survival analysis includes data from all programs that had at least one start date for any activity (*n* = 34). From the survival analysis, the median predicted implementation time to statewide screening is 75 months (95% CI = 45, 99) for Pompe and 66 months (95% CI = 33, 75) for MPS I. For ALD, the largest observation was censored, and the estimation was restricted to the largest event time (Figure 1). There was no significant difference in time to implementation between Pompe, MPS I, and ALD (Log-Rank χ^2^ (df 2) = 2.16, *p* = 0.34).

While statewide implementation is important, various readiness activities were still in progress and/or completed after NBS programs began statewide screening of a new disorder. At least one of the 17 NBS programs who had reached statewide screening was working towards completing an activity post-implementation across all phases except for approval to screen. When looking at activities completed post-implementation for at least two disorders, follow-up readiness is dropped because it is only identified for MPS I (Table 2). The majority of programs’ education activities were still in progress at the time of statewide screening, with “identifying/creating measures to track the impact of educational materials” as the most frequently reported post-implementation activity (Table 2). 

### 3.2. Time from Approval/Mandate to Implementation of Statewide Screening

To predict time needed for states to begin screening for these disorders prior to addition to the RUSP, the Public Health Impact Assessment (PHIA) asked states to estimate how long it will take to implement statewide screening once they received approval to screen. Therefore, the actual time between approval for screening and implementation of statewide screening was examined for states who provided both completion dates (Pompe *n* = 9; MPS I *n* = 11; ALD *n* = 5; and SMA *n* = 2). For these states, it took a median observed time of 5.5 to 16 months to begin statewide screening once the program obtained approval (Figure S2). 

### 3.3. Time to Complete Authority to Screen and Program Readiness (Laboratory/Follow-Up/IT) in the Implementation Process

In addition to understanding the time to statewide implementation for Pompe, MPS I, ALD, and SMA, it is important to understand how long each phase takes from start to completion. Laboratory readiness was the longest phase for Pompe (observed median 23 months; *n* = 14) and MPS I (observed median 24 months; *n* = 14). Implementing a fee increase was the longest phase for ALD (observed median 21 months; *n* = 13) and SMA (observed median 22 months; *n* = 5). Laboratory readiness and implementing a fee increase are still the longest phases in the survival analysis (predicted median), but there are some differences in the longest phases when comparing the observed medians to the predicted medians. The longest predicted median time-shifted to implementing a fee increase for MPS I (predicted median 49 months; *n* = 30) and ALD (predicted median 27 months; *n* = 30). Lab readiness was still the longest phase for Pompe (predicted median 39 months; *n* = 28). All predicted median times were longer than the observed median time reported by states who had completed these phases (Table 3).

### 3.4. Understanding Which Readiness Phase Activities Take the Longest Time to Complete

When looking at the top five activities that took the longest to complete for each disorder based on the beginning and end dates reported, developing/validating an assay for first-tier testing came up the most often, taking an observed median time of just over a year for Pompe, MPS I, and ALD (Table 4). The most time-consuming activities for SMA were approval to screen and approval for funding to screen. The longest observed median time for a single activity was developing/validating an assay for second-tier testing to improve the specificity of the screen for ALD. It should be noted that the meidan time for these longer steps were aQfor Pompe, MPS I, and ALD and that they only represent states who had completed the activity. 

### 3.5. Time from Addition to RUSP to Initiation of First Activity

Equally important to knowing how long readiness activities take is understanding how long it takes NBS programs to begin implementation activities once the disorder has been added to the RUSP. Excluding states that started the implementation process prior to the disorder being added to the RUSP, it took between 3 and 23 median months until initiation of the first activity from the RUSP addition date. Pompe was the only new disorder where no program started working on a readiness activity immediately (Figure S3).

### 3.6. Barriers and Facilitators to Implementing Statewide Screening

**Figure 2:**
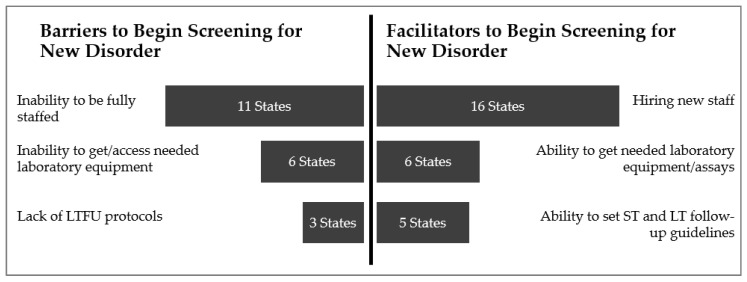
Factors Identified as both Barriers and Facilitators to Implementation of Statewide Newborn Screening of a New Disorder, Collected from Grantee Progress Reports and Interviews.

Facilitators and barriers were identified by the 16 NewSTEPs New Disorder program awardees. These facilitators and barriers could be confounders in identifying the difference in time to implementation between the state NBS programs. Issues such as staffing, obtaining necessary laboratory equipment, and establishing follow-up protocols were both barriers and facilitators amongst the awardees (Figure 2). There were no differences in the barriers or facilitators mentioned by disorder, but most states were working on implementing multiple disorders.

In terms of staffing as a barrier, newborn screening programs mentioned staff shortages of both laboratory staff (*n* = 9/16) and follow-up staff (*n* = 3/16).

“[Program is] not making headway on training and keeping new staff. There are no simplified reviews for new positions. It is needlessly hard to hire.”

“Getting well-qualified people with what the state pays is the challenge. [We] get them green, train them, and they leave.”

Barriers around equipment included not having FDA-approved laboratory kits for testing or equipment (*n* = 3/16), difficulty optimizing new equipment (*n* = 3/16), and equipment failure (*n* = 3/16). While states are not required to have FDA-approved laboratory kits for testing, it can be easier for some states to use these kits than develop their own tests for the disorder.

One final barrier identified is the lack of institutional knowledge about how to prepare for screening for a new disorder because so few NBS programs had knowledge or experience. “Our timelines are longer because we were the first [program] and had so much to validate before we could start our full-population pilot.”

Collaboration amongst stakeholders was identified as a facilitator. Several awardees described state newborn screening program workgroups, which included consumers, geneticists, follow-up, and laboratory specialists (*n* = 7/16) as helping increase the state’s readiness to implement statewide screening.

“[Our program is] given six months after getting equipment to begin screening. During this time, we meet with vendors; ask follow-up, laboratory, specialists, and the [advisory group] to work together on what needs to be reported out and how to treat special baby population populations. Decisions are made on what to do for different testing results and special groups during this time [through collaboration].”

“[To] create education materials for the new disorders, [we] used the lysosomal disorder workgroup..., had a neonatologist, parent, people from laboratory and follow up on workgroup. [We also] had a pediatrician consultant help. [The workgroup] gave edits and comments. When [the education materials were] approved [program leadership] took it before the director and the commissioner and got final approval with it.”

There was also cross-collaboration amongst states (*n* = 9/16 identified this facilitator). Collaboration outside the state newborn screening program included working with Peer Network Resource Centers (PNRCs), as well as programs that were further along in the implementation process to help with testing and validation, “shadowing,” a program to better understand workflow and staffing roles, sharing resources, adapting education materials created by other states or national programs like Baby’s First Test (https://www.babysfirsttest.org/), or the National Institutes of Health (https://www.nichd.nih.gov/health/topics/newborn/more_information/resources).

## 4. Discussion

This study provides the first-time estimate to implement a newborn screening system for new disorders using start and completion dates collected from participating states. Thirty-nine states provided data on Pompe, MPS I, ALD, and/or SMA. Historically, the only estimate of time needed for implementing statewide screening for a new disorder is based on data gathered as part of the PHIA conducted by APHL for ACHDNC [6,11,12,13]. Readiness Tool data are in alignment with the PHIA for states who have completed the processes to implement a new disorder, but differ when we incorporate those that are still working towards implementation, suggesting a bias is introduced when only evaluating those states that are able to implement early.

### 4.1. Time to Statewide Screening

We found wide variation in how long it takes to implement statewide screening for the four disorders, and accounting for programs that were still in progress is critical in accurately estimating the time required. Incorporating the censored data reveals that the predicted median is twice as long as the observed median time. The 17 participating states who began statewide screening for any of the four new disorders before 31 August 2019 had an observed median time ranging from 20 to 30.5 months to implement at least one new disorder. However, the majority of states were still in progress towards implementation. By using the survival analysis to include those who could provide a start date for at least one activity, whether or not they had implemented statewide screening, states were predicted to take a median of 66 to 75 months.

The variation in the time needed to implement statewide screening is multifaceted. The largest median time for a specific readiness phase for MPS I and ALD was mandate/approval to screen, with a predicted range of eighteen months to four years. The variation may be due to differences in the number of steps in the approval process for a given jurisdiction. Some states do not need regulation changes to add a condition, while others have a multi-stage legislative or approval process [17,18]. One interviewee explained that in their state, new disorders are reviewed by four committees prior to making a request to change the regulations, which then is subjected to public comment period prior to approval. The legislative step may be even slower in states with “part-time” legislatures—state legislative bodies that meet for as little as 30 days a year [18]. While the approval to screen process is slow, the interviewee explained that there is a benefit in giving the newborn screening program enough time to “systematically and carefully” introduce the new disorder to the state panel.

Another phase that was predicted to take over three years to complete was laboratory readiness for Pompe and MPS I, which is impacted by the screening test for the disorder being added. Developing and validating assays can take 12 to 20 months. Adding conditions whose testing can be performed on instrumentation already in use by newborn screening programs allows states to complete this step faster if they do not need to purchase additional equipment or modify their laboratory space [18]. States reported that identifying and obtaining new laboratory equipment can take about a year if there is space, while modifying space for the laboratory equipment can take an additional 17 months. Not having FDA-approved laboratory kits for testing was identified as a barrier, but no data were collected on the timing for a manufacturer to bring a test to market after a condition is added to the RUSP. Future research should look at the average time and, if needed, how that process can be shortened.

For states that were able to provide both start and completion dates for implementation activities, the most time-consuming activity was developing/validating an assay for 2nd tier testing for ALD. Please see Table S1 for the types of 2nd and 3rd tier testing methodolgogies reported by states for each disorder. An interviewee explained the positive impact of 2nd tier testing on the workflow for follow-up: “For MPS I and Pompe [we] love 2nd tier. [Our newborn screening program is] saving time not picking up pseudo-deficiencies.”

Being ready to screen only at the laboratory level is not sufficient. Developing follow-up protocols and ensuring adequate staffing were two of the most time-intensive readiness steps for Pompe and ALD; for Pompe, these activities took the longest. Some interviewees identified not having long-term follow-up protocols as a barrier, while other interviewees noted being able to set short-term (ST) and long-term follow-up (LTFU) guidelines was a facilitator for screening. As one interviewee noted:

“[It is] easy and cheap to add SMA, but [testing for SMA] puts follow-up in a tough situation sometimes. [Our program has] specialists who don’t know what happens yet, [don’t know] what questions they have yet. [Newborn screening program] is not ready to bring a child through the process [of having positive newborn screen], especially if [the screen] is a false positive?”

This quote also highlights the importance of provider education prior to statewide testing. Another interviewee illustrated how educating providers can help improve the newborn screening program’s workflow. “Provider education is important. When [we] first started screening LSDs [lysosomal storage disorders]. [We] asked for repeats based on gestational age or birth weight, but then stopped because learned [it is] not really an issue in their test. [Follow-up] spent a lot of time tracking down repeats that were not needed.”

### 4.2. Time from Addition of the RUSP to Statewide Screening

While time from the first activity to statewide screening is important, not all programs can begin the process of adding the new disorder to the state panel immediately following the addition to the RUSP. We found that for states who did not start any readiness activities prior to the condition being added to the RUSP, it could take 5 to 11 months to begin the implementation process. This is much lower than what past studies have found. One study reported that it took states a median of 4.4 years to adopt Severe Combined Immuodeficiency ( SCID) on their state newborn screening panel and a median of 3.2 years for Critical Congenital Heart Disease (CCHD) [18]. These authors stated that the RUSP expansion has led to slower adoption of the new condition. In our study, an interviewee supported this and explained, “[We] don’t want to bring on too many conditions at once because it is a burden for the specialists...they are the program’s go-to for diagnosis and referral network... so don’t want to overwhelm them.”

When asked about the delay, interviewed states mentioned not having access to the resources needed, spanning across the NBS system:

“The primary reason we put testing on hold is because we didn’t have the reagent to screen for it [ALD].”

“We did have staff, but we did not have instrument time. [We had] an agreement [with another agency to access their lab instrumentation], but by [the time of the addition to the RUSP for] ALD, they had too little [available instrument] time to help.”

In some states, staffing was identified as the biggest barrier, while in others, it was the biggest facilitator. This is not surprising, as we have previously described the challenges state newborn screening programs have in hiring and retaining staff [19]. Programs can identify strategies that may be shared between programs; however, implementing solutions can be challenging. While some programs continue to seek solutions to short- and long-term staffing challenges, most programs seek collaboration with other programs that have implemented already; the PNRC is a key example of this type of work. Collaboration with other newborn screening programs was the second most frequently mentioned facilitator. Specifically, sharing resources for screening algorithms, follow-up protocols, and education materials were identified as helping the readiness process go more smoothly in a state that adopted the disorder later compared to early adopters.

### 4.3. Limitations

As with all studies, there were some limitations. While some states were able to provide exact start and completion dates, many dates were estimated or rounded to a certain month and year. This is why we decided to report median months vs. median days. Further, some NBS programs completed the activities so long ago, staff could not remember when or how long an activity took, leaving start and completion dates missing, thus limiting the sample size for analysis. Although the Readiness Tool was created in collaboration with NBS programs, some steps to the implementation process may have been missed or over-simplified. For instance, some interviewees noted that the Readiness Tool needs to include more specific steps related to follow-up readiness in order to accurately measure phase completion.

There are a number of states who did not complete the Readiness Tool because they had implemented statewide screening early and could not remember the time frames, they were not ready to implement screening, or they were not interested in providing the data. This selection bias could underestimate or overestimate how long statewide implementation truly takes. Our findings are only reflective of the 39 states who did provide data.

The Readiness Tool did not capture data around the use of quality assurance administered by the Centers for Disease Control and Prevention (CDC) or creating lab-developed tests (LDTs). Some states cannot implement statewide screening for a new disorder until they can meet their Clinical Laboratory Improvement Amendments/College of American Pathologist (CLIA/CAP) requirements for patient testing, and not having the quality assurance materials to do this may slow down implementation. Future research on the time to full implementation of a new disorder should gather the time involved in this readiness step.

Finally, it should be noted that SMA was not on the RUSP when the project started. As a result, the data for this condition may reflect the early adopters. Towards the end of the project, newborn screening programs were asked to complete the Readiness Tool for SMA if possible. Only 30 states were able to provide any data.

## 5. Conclusions

Based on our findings, it may take states around five to six years to be able to implement statewide screening for Pompe, MPS I, ALD, and SMA. As is evident by this analysis and the time to statewide implementation analysis, states are working on the different readiness phases concurrently. However, there is a lot of variation due to differences in state approval process, ability to obtain required equipment, having dedicated staff, and availability of needed resources for adding the condition to state NBS panels. The ability to get ready to screen may also be hampered by the number of new disorders being added to the RUSP. All of these potential barriers may be hard for states to estimate how much time they will need to implement statewide screening that includes follow-up readiness and education readiness. Future research may want to examine if there is a critical point in each phase that impacts the time needed for statewide implementation of testing. Future research should also look at the impact of NBS on long-term prognosis for children diagnosed through NBS. The results of this project can start a broader discussion of what support could be most beneficial to NBS programs as they work to expand the number of disorders screened for in their communities.

## Figures and Tables

**Table 1:** Implementation Status of Participating Newborn Screening Programs Collected from the New Disorder Readiness Tool, as of August 2019.

	Number Who Provided Data (n)	Statewide Screening Implemented	In Progress Towards Statewide Screening (i.e., Completed at Least One Activity)	Not Yet Started an Activity	Not Actively Pursuing Statewide Screening
**Pompe**	39	11 (28.2%)	23 (59.0%)	4 (10.3%)	1 (2.6%)
**MPS I**	38	13 (34.2%)	21 (55.3%)	4 (10.5%)	0 (0.0%)
**ALD**	38	8 (21.1%)	26 (68.4%)	3 (7.9%)	1 (2.6%)
**SMA**	30	5 (16.7%)	20 (66.6%)	2 (6.6%)	3 (10.0%)

**Table 2:** Readiness Activities Completed Post-Implementation * of Statewide Newborn Screening that Occurred for at least Two New Disorders; Collected from the New Disorders Readiness Tool, as of August 2019.

Readiness Phase	Activity	Pompe (*n* = 11)	MPS I (*n* = 13)	ALD (*n* = 8)	SMA (*n* = 5)
**Approval for Funding**	Fee Increase Implemented	1 (9%)	1 (8%)	0 (0%)	1 (20%)
**Laboratory Readiness**	Add Testing to Outside Laboratory Contract	2 (18%)	1 (8%)	0 (0%)	0 (0%)
	Train Laboratory Staff to Begin Testing	1 (9%)	1 (8%)	1 (13%)	1 (20%)
**IT Readiness**	Validate Changes to the Follow-up Reporting System	0 (0%)	2 (15%)	0 (0%)	1 (20%)
	Validate Changes to Electronic Results Protocol	0 (0%)	1 (8%)	0 (0%)	1 (20%)
	Other IT Activities (e.g., getting DNA sequencing into IT; changing LIMS)	1 (9%)	1 (8%)	1 (13%)	0 (0%)
**Education**	Identify/Modify Family Education Materials to be State Specific	0 (0%)	1 (8%)	2 (25%)	1 (20%)
	Initiate a Strategy for Family Education Materials & Create Own Family Education Materials	1 (9%)	2 (15%)	1 (13%)	1 (20%)
	Identify/Create Measures to Track Impact of Family Education Materials	5 (46%)	6 (46%)	2 (25%)	2 (40%)
	Family Education Materials are Distributed	1 (9%)	2 (15%)	0 (0%)	1 (20%)
	Identify/Modify Provider Education Materials to be State Specific	0 (0%)	1 (8%)	1 (13%)	0 (0%)
	Identify/Create Measures to Track Impact of Provider Education Materials	6 (55%)	6 (46%)	2 (25%)	2 (40%)
	Identify/Modify General Public Education Materials to be State Specific	1 (9%)	2 (15%)	0 (0%)	1 (20%)
	Initiate a Strategy for General Public Education Materials & Create Own General Public Education Materials	1 (9%)	2 (15%)	0 (0%)	1 (20%)
	Identify/Create Measures to Track Impact of General Public Education Materials	6 (55%)	7 (54%)	2 (25%)	1 (20%)
	General Public Education Materials are Distributed	1 (9%)	2 (15%)	0 (0%)	1 (20%)

* Activity may have started prior to implementation but did not end until after statewide implementation.

**Table 3:** Comparison of Observed Median Time and Predicted Median Time Estimated using Kaplan–Meier Survival Analysis to Complete Each Phase of the Implementation Process for Statewide Newborn Screening for New Disorders, as of August 2019.

		Mandate/Approval to Start Screening	Fee Increase Implemented	Laboratory Readiness	Follow-Up Readiness	IT Readiness
Predictive Median Months from Survival Analysis [95% CI] (number of state NBS programs)	Pompe	NA ^+^	24	39	26	16
		(27) ^β^	(15) ^β^	(24,68)	(18,36)	(7,37)
		(*n* = 25)	(*n* = 27)	(*n* = 28)	(*n* = 24)	(*n* = 19)
	MPS I	36 *	49 ^**^	39	24	18
		(18) ^β^	(22,49)	(23,60)	(13,42)	(7,37)
		(*n* = 25)	(*n* = 30)	(*n* = 28)	(*n* = 24)	(*n* = 20)
	ALD	NA ^+,^*	27	NA ^+^	NA ^+^	14
		(17) ^β^	(24) ^β^	(31) ^β^	(26) ^β^	(7) ^β^
		(*n* = 24)	(*n* = 30)	(*n* = 30)	(*n* = 26)	(*n* = 18)
Observed Median Months to Complete Phase (Min, Max) (number of state NBS programs)	Pompe	18.0	16.5	23.0	21.5	12.5
		(9,30)	(4,34)	(4,68)	(1,77)	(2,40)
		(*n* = 9)	(*n* = 14)	(*n* = 14)	(*n* = 14)	(*n* = 14)
	MPS I	13.0 *	19.5 **	24.0	18.5	13.5
		(3,36)	(4,49)	(14,75)	(5,42)	(2,46)
		(*n* = 11)	(*n* = 12)	(*n* = 14)	(*n* = 14)	(*n* = 14)
	ALD	12.0 *	21.0	20.0	18.0	8.0
		(0,30)	(1,34)	(13,36)	(2,30)	(2,14)
		(*n* = 9)	(*n* = 13)	(*n* = 9)	(*n* = 9)	(*n* = 9)

Table excludes newborn screening (NBS) programs that selected “NA” for phase completion variables or did not provide start and/or completion dates. Spinal Muscular Atrophy (SMA) was not included as not enough time had elapsed from its inclusion on the Recommended Uniform Screening Panel (RUSP) to the end of data collection to allow for accurate estimates. Further, education was removed because few states have completed this phase. ^+^ The predicted median time could not be calculated because more than 50% of participating programs did not complete the phase. ^β^ Upper bound of 95% confidence intervals (CI) could not be calculated because the largest observation was censored. * One NBS program is excluded from analysis because the mandate to screen for MPS I and ALD occurred prior to the start of the phase (i.e., NBS Advisory Committee Approval). ** One NBS program is excluded from Fee Increase Implementation for MPS I as their start date occurred after their completion date.

**Table 4:** Top Five Newborn Screening Implementation Activities with the Longest Observed Median Time to Complete, Collected from the New Disorders Readiness Tool, as of August 2019.

Longest to Shortest	**Pompe** **(Median Months, *n*)**	**MPS I** **(Median Months, *n*)**	**ALD** **(Median Months, *n*)**	**SMA** **(Median Months, *n*)**
	Develop follow-up staffing protocols/ensure adequate staffing (12.6, *n* = 13)	Identify screening methodology/assay for 1st tier testing (14.8, *n* = 15)	Develop/validate assay for 2nd tier testing (19.7, *n* = 3)	Obtain approval from the state budget authority (10, *n* = 6)
	Develop/validate assay for 1st tier testing (12.4, *n* = 12)	Other funding activities * (14.8, *n* = 4)	Modify laboratory space; install equipment (17.4, *n* = 8)	Other approval/authority activities (7.8, *n* = 4)
	Identify/modify general public education materials to be state-specific (12.0, *n* = 8)	Develop/validate assay for 1st tier testing (12.5, *n* = 14)	Develop/validate assay for 1st tier testing (12.7, *n* = 6)	Ensure adequate laboratory space for testing (6.4, *n* = 12)
	Identify screening methodology/assay for 1st tier testing (11.9, *n* = 13)	Procure additional laboratory equipment (11.8, *n* = 13)	Other funding activities (12.7, *n* = 3)	Develop budget to show cost for screen (6.2, *n* = 12)
	Identify/modify family education materials to be state-specific (11.6, *n* = 8)	Identify/modify general public education materials to be state-specific (11.7, *n* = 9)	Identify medical specialists/treatment center (11.9, *n* = 13)	Obtain approval from NBS Advisory Committee (5.3, *n* = 15)

Activities listed in this table are based on the number of newborn screening (NBS) programs that selected “completed” and provided start and end dates. * Other funding activities include activities such as approval for use of specific funds for pilot testing and applying for and/or receiving external funds to cover cost to implement statewide screening.

## Supplementary Materials

The following are available online at https://www.mdpi.com/2409-515X/6/2/35/s1.
