# Supplementary material for: Implementing Statewide Newborn Screening for New Disorders: U.S. Program Experiences
Source: Int J Neonatal Screen. 2020 Apr 30;6(2):35. doi: 10.3390/ijns6020035 (PMC7422992; doi:10.3390/ijns6020035)
Supplement: Supplementary file 1 [file IJNS-06-00035-s001.pdf]

Supplemental Graphs for Implementing Statewide Newborn Screening for New Disorders: US Program Experiences

By

Yvonne Kellar-Guenther [1,2,*], Sarah McKasson [3], Kshea Hale [4], Sikha Singh [4], Marci K. Sontag [1,3], and Jelili Ojodu [4]

1 Center for Public Health Innovation, CI International, 7852 S. Elati St., #201, Littleton, CO 80120, USA; ykellar-guenther@ciinternational.com (Y.K.G); msontag@ciinternational.com (M.K.S.)
2 Colorado School of Public Health, Department of Community and Behavioral Health, University of Colorado, Anschutz Medical Campus, Aurora, CO 80045, USA; Yvonne.kellar-guenther@cuanschutz.edu (Y.K.G.);
3 Colorado School of Public Health, Department of Epidemiology, University of Colorado, Anschutz Medical Campus, Aurora, CO 80045, USA; sarah.mckasson@cuanschutz.edu (S.M.), msontag@ciinternational.com (M.K.S.);
4 Association of Public Health Laboratories, 8515 Georgia Avenue, Suite 700, Silver Spring, MD 20910, USA; kshea.hale@aphl.org (K.H.); sikha.singh@aphl.org (S.S.); jelili.ojodu@aphl.org (J.O.)
* Correspondence: ykellar-guenther@ciinternational.com; Tel.: +1-303-829-0819

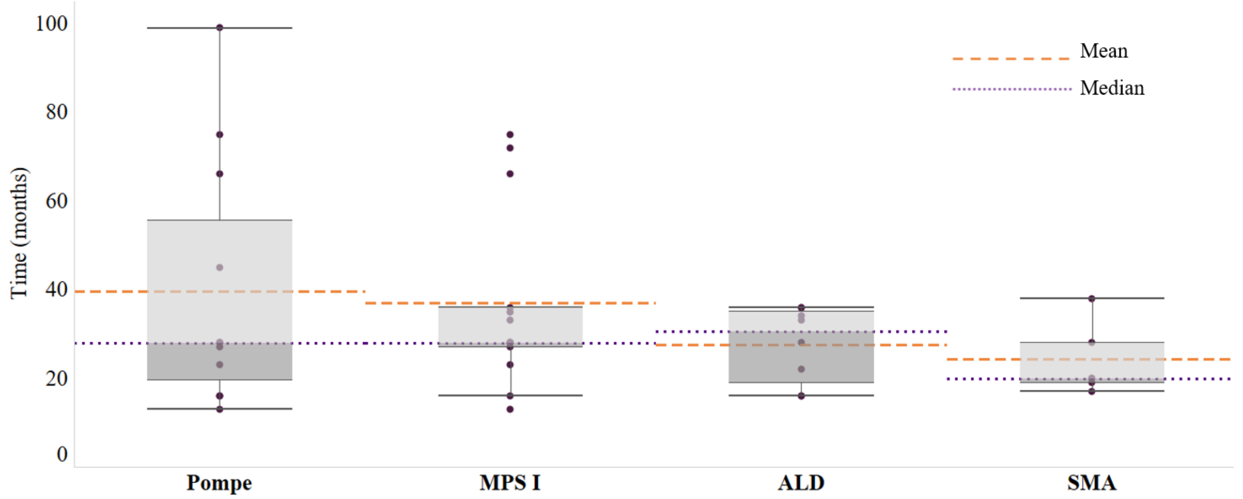

	Number (n)	Mean Time (months)	Median Time (months)	Minimum Time (months)	Maximum Time (months)
**Pompe**	11	39.64	28.00	13	99
**MPS I**	13	36.92	28.00	13	75
**ALD**	8	27.63	30.50	16	36
**SMA**	5	24.40	20.00	17	38

**Time is calculated from the earliest start date provided in the New Disorder Readiness Tool to the implementation date for statewide screening.*

Figure S1: Time from Initiation of First Activity to Implementation of Statewide Newborn Screening for New Disorders

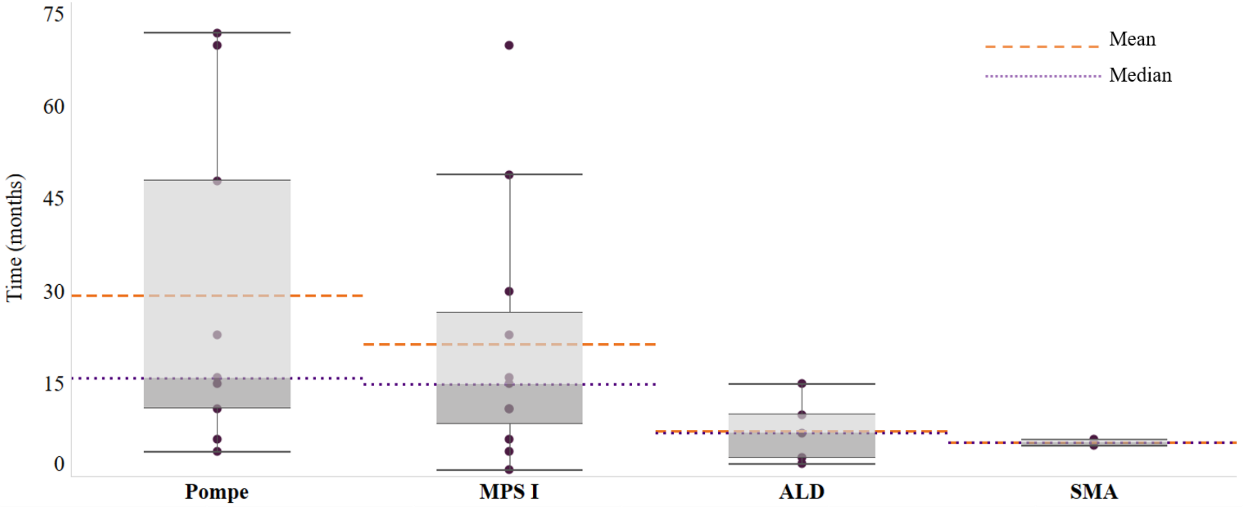

	Number (n)	Mean Time (months)	Median Time (months)	Minimum Time (months)	Maximum Time (months)
**Pompe**	9	29.44	16.00	4	72
**MPS I**	11	21.45	15.00	1	70
**ALD**	5	7.40	7.00	2	15
**SMA**	2	5.50	5.50	5	6

Figure S2: Time from Approval/Mandate to Screen to Implementation of Statewide Newborn Screening for New Disorders

Figure S2: Time from Approval/Mandate to Screen to Implementation of Statewide Newborn Screening for New Disorders

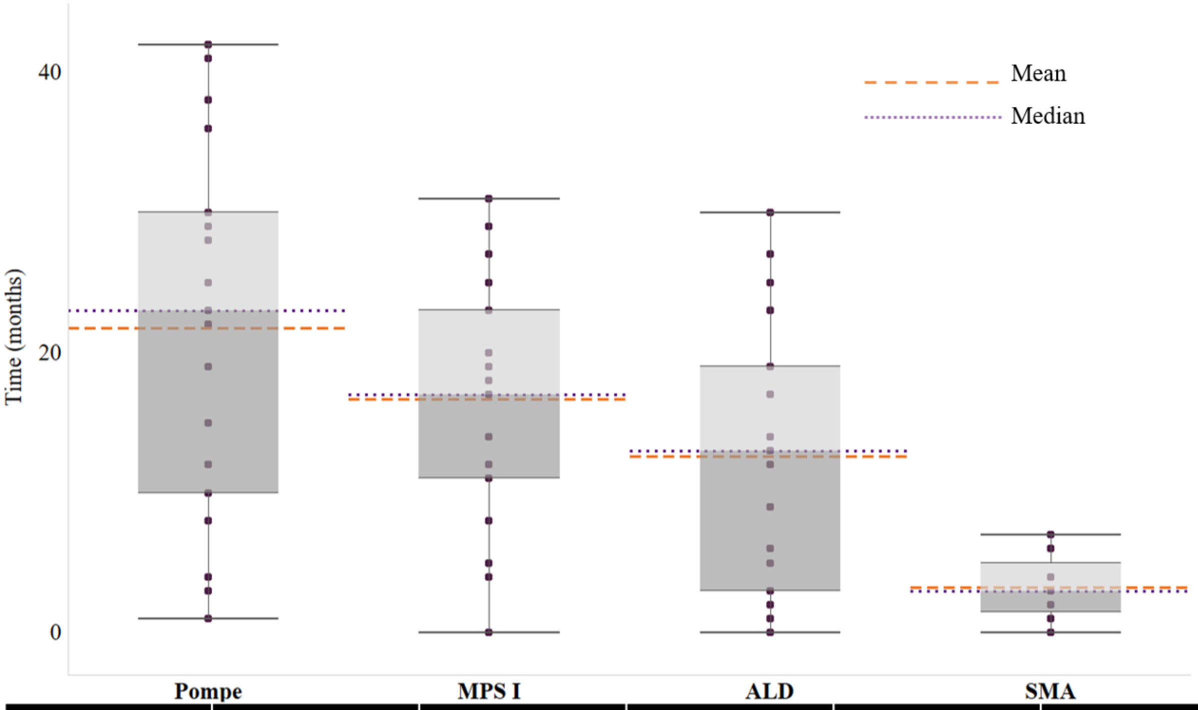

	Number (n)	Mean (months)	Median (months)	Min (months)	Max (months)
**Pompe**	22	21.78	23.00	1	42
**MPS I**	20	16.67	17.00	0	31
**ALD**	19	12.63	13.00	0	30
**SMA**	8	3.25	3.00	0	7

**Time is calculated from the data disorder was added to the RUSP to the earliest start date entered in the New Disorder Readiness Tool. Figure excludes programs that started an activity prior to the disorder being added to the RUSP.*

Figure S3: Time from Addition of New Disorder on the RUSP to Initiation of First Activity taken by State Newborn Screening Program to Move towards Implementation of Statewide Screening for the Disorder.

Table S1. Types of 2nd and 3rd Tier Testing Reported by States for each Disorder, Collected from the New Disorder Readiness Tool, August 2019.

		MS/MS	LS-MS/MS	Digital Microfluidics	Molecular Methodology	Other*	Missing
2nd tier	Pompe (n=16)	3	0	0	9	1	3
	MPS 1 (n=16)	1	2	0	7	3	3
	ALD (n=17)	0	11	0	1	0	5
	SMA (n=4)	0	0	0	2	0	2
3rd tier	Pompe (n=3)	0	0	0	3	0	0
	MPS 1 (n=4)	0	0	0	3	0	1
	ALD (n=8)	0	0	0	7	0	1

*Other 2nd tier testing for Pompe included FIA-MS/MS, Creatine, Creatinine, activity of acid-alpha glucosidase (GAA) and other LSD enzymes interpretation by CLIR tool. Other 2nd tier testing for MPS I included LC-MS/MS send out to Mayo Medical Laboratories (MML); biochemical test for heparan sulfate, dermatan sulfate, and keratan sulfate; GAG analysis on DBS; and Genetic sequencing of gene at reference lab.
